# Development and Characterization of High-Density Polyethylene/Polylactic Acid/Titanium Dioxide Composites for Pellet-Based 3D Printing

**DOI:** 10.3390/polym18121475

**Published:** 2026-06-12

**Authors:** Ildiko Peter, Dan-Cristian Craciun, Mihai Alin Pop

**Affiliations:** 1 Department of Industrial Engineering and Management, Faculty of Engineering and Information Technology, George Emil Palade University of Medicine, Pharmacy, Science, and Technology of Targu Mures, 540142 Targu Mures, Romania; ildiko.peter@umfst.ro; 2 Doctoral School of I.O.S.U.D., George Emil Palade University of Medicine, Pharmacy, Science, and Technology of Targu Mures, 540142 Targu Mures, Romania; 3 Materials Science Department, Transilvania University of Brasov, 500036 Brasov, Romania; mihai.pop@unitbv.ro

**Keywords:** high-density polyethylene, polylactic acid, titanium dioxide, pellet-based 3D printing, thermal degradation, polymer composites

## Abstract

In the present study, the development of a high-density polyethylene/polylactic acid/titanium dioxide (HDPE–PLA–TiO_2_) composite proposed for pellet-based additive manufacturing and the evaluation of its thermal and mechanical behavior are presented and discussed. The study was designed to address the printability limitations of high-HDPE-content systems, particularly extrusion instability and weak interlayer adhesion. PLA was introduced to improve processing stability, while TiO_2_ was incorporated as an inorganic filler. The selected formulation allowed the production of filaments, pellets, and 3D-printed specimens. Thermal analysis indicated the absence of significant mass loss below approximately 300 °C under the applied thermogravimetric/differential thermal analysis (TG/DTA) conditions, suggesting that no major mass-loss degradation occurred within the selected processing window. However, this result should be interpreted as macroscopic thermal stability and does not exclude possible molecular-level changes in PLA during processing. Tensile tests indicated strengths of 20–25 MPa for extruded filaments and 7.86–10.36 MPa for printed specimens, with an elastic modulus of approximately 2 GPa. Scanning Electron microscopy equipped with Energy Dispersive X-Ray Spectroscopy (SEM/EDS) observations revealed a heterogeneous fracture morphology with cavities, microcracks, fibrillar structures, and local Ti-rich regions, supporting the influence of morphology and filler distribution on the mechanical response of the printed specimens. The results indicate improved printability, adequate thermal behavior for the selected processing conditions, and moderate but reproducible tensile performance, highlighting the potential of this formulation for pellet-based additive manufacturing applications where processability and rigidity are more relevant than maximum tensile strength.

## 1. Introduction

Generally, polymers are used in different industrial areas, including the automotive, electrical, construction, packaging, and medical industries, due to their low density, versatility, and ease of processing. In order to be used in a wider range of fields, an improvement in their properties is needed, which is why they are mixed with additives or reinforced with other materials that lead to the improvement of some properties, such as stiffness, dimensional stability, rigidity, etc. [1,2].

Additive manufacturing (AM) is an important process in the development of polymeric materials and their composites, due to its flexibility, reduced costs, and the possibility of customized fabrication. Among AM processes, material extrusion (MEX) is the most widely used due to its compatibility with thermoplastic polymers and the flexibility in fabricating parts with controlled internal structures and geometries. Fused filament fabrication (FFF) remains one of the most used MEX technologies, but recently pellet-based extrusion, fused granulate fabrication (FGF), has gained importance because it allows the direct processing of materials in the form of pellets, thus replacing the classical filament. This approach is particularly useful for recycled polymers, polymer blends, and composite feedstock materials, which are increasingly used in sustainable manufacturing routes, while recycling has become a necessity. In this way, filament fabrication can be reduced and more sustainable manufacturing routes, which are more efficient from the cost point of view, can be supported [3,4,5,6,7,8,9,10]. Recent studies confirm the increasing relevance of screw-extrusion FGF and additive manufacturing using pellets for recycled polymeric systems or blends containing HDPE, which are processed directly from pellets [6,11]. The quality and performance obtained through FFF or FGF processes depend entirely on processing parameters, such as nozzle temperature, feeding stability, interlayer bonding, and porosity formation [4,5,9,12].

A thermoplastic widely used due to its low density is HDPE; it presents chemical resistance, durability, and industrial availability. HDPE is characterized by low polarity and low reactivity, with the more reactive regions being the terminal bonds and the tertiary C–H bonds, which are strongly influenced by the processing conditions and by the thermal history [13,14,15,16]. This material is nevertheless difficult to process by extrusion-based additive manufacturing compared with more frequently used polymers, such as PLA. HDPE-rich systems often present pronounced shrinkage, deformation, limited adhesion during printing, weak bonding between layers, and dimensional instability during printing, difficulties that reduce the reproducibility and mechanical performance of the created parts. This is the barrier to the use of HDPE in pellet-based 3D printing, so that the development of HDPE-based formulations with improved processability remains an important research direction for the additive manufacturing of polymers [6,11,15,16].

Previous studies on HDPE-based systems have shown that the final mechanical behavior is strongly influenced by processing conditions, material formulation, and reinforcement strategy. Improved mechanical performance is reported in joints or composites when the processing conditions and the reinforcement effects were carefully controlled [17,18].

One of the most used thermoplastics for extrusion-based 3D printing is PLA, due to its good rigidity, relatively high strength, dimensional stability, and ease of processing. PLA is a biodegradable polymer obtained from renewable resources and has been widely investigated for applications in packaging, disposable products, textiles, and additive manufacturing [19,20,21,22,23,24,25]. Processability, structural characteristics, and possible modifications have been extensively discussed in the specialized literature, confirming its relevance for polymer processing and composite development [26,27,28,29,30,31,32]. In HDPE-rich composites, PLA can act as a secondary polymeric phase, which not only improves melt processability, but also increases rigidity, and achieves a more stable extrusion. It is also well known that the two polymers have low compatibility due to their different polarities and molecular structures; this denotes that any improvement is interpreted as a processing advantage of the selected formulation, rather than as evidence of complete interfacial compatibility between the two phases [33,34].

An inorganic filler widely investigated and used in polymer composites due to its chemical stability is TiO_2_; also, it is cost-effective, provides ultraviolet (UV) radiation screening, and contributes to the thermal residual fraction [3,35,36,37]. Through the incorporation of TiO_2_ into polymer matrices, certain physical and functional properties can be improved. Numerous studies have investigated HDPE-based composites, PLA-based materials, polymer composites containing TiO_2_, and the processing of recycled polymers through FFF or FGF; however, the available information on HDPE–PLA–TiO_2_-rich composites developed specifically for pellet-based 3D printing is limited. The existing studies are often dedicated either to HDPE–TiO_2_ MEX filaments, or to PLA-based systems, or recycled blends containing HDPE, or general FGF processing strategies [3,6,9,11,12]. The combined stages involving composite extrusion, pellet preparation, and subsequent pellet-fed 3D printing of an HDPE–PLA–TiO_2_-rich system remain insufficiently discussed. This gap is relevant because the printability of materials with a high degree of HDPE content depends not only on the chemical composition, but also on the interaction between extrusion stability, pellet feeding, melt flow, and deposition behavior [3,6,9,12].

The present study should be interpreted as a preliminary optimization and thermo-mechanical characterization of an HDPE-rich multiphase composite developed for pellet-based additive manufacturing. The aim was not to provide a complete molecular or microstructural validation of the HDPE–PLA–TiO_2_ system, but to evaluate whether the proposed formulation can improve extrusion stability, pellet-fed printability, and the basic thermal and tensile response of printed specimens. Therefore, the results are discussed within the limits of the performed experimental program, with an emphasis on processability, thermal mass-loss behavior, and mechanical reproducibility under the selected printing conditions.

## 2. Materials and Methods

### 2.1. Main Materials

High-density polyethylene (HDPE 5502BN) supplied by Electric SRL, Focșani, Romania, was used as the main polymer base. According to the manufacturer’s datasheet, the material has a density of 0.955 g/cm^3^, a melting temperature of 190 °C, and an elastic modulus of 1.2 GPa. Recycled polylactic acid (PLA) filament supplied by Spectrum, Poland, with a nominal diameter range of 1.75–2.85 mm, was used as a secondary polymeric component in order to improve the processability and printability of the HDPE-based system. Titanium dioxide (TiO_2_) in granular form, supplied by Indra Import-Export, was used as an inorganic filler to reduce UV radiation absorption during the service life of the material. The final composite formulation consisted of 58 wt.% HDPE, 40 wt.% PLA, and 2 wt.% TiO_2_.

### 2.2. Composite Formulation

The composite material was designed using HDPE as the major component, while PLA and TiO_2_ were incorporated to improve extrusion stability and printability. During the optimization stage, several formulations were investigated in order to reduce the volumetric expansion of HDPE during extrusion and to obtain a more homogeneous material. The final formulation was found to be suitable for 3D printing, ensuring improved dimensional stability and preventing excessive volumetric expansion during the extrusion process.

The chemical composition of the developed material is simple, consisting of 58% HDPE, 40% PLA, and 2% TiO_2_. The manufacturing process follows the workflow below. The process begins with extrusion using the equipment, under the working parameters specified in Section 2.3. After the filament is produced, pellets of 2–4 mm are prepared, which are subsequently introduced into the 3D-printing equipment for the fabrication of the actual test specimens.

**Scheme 1:**
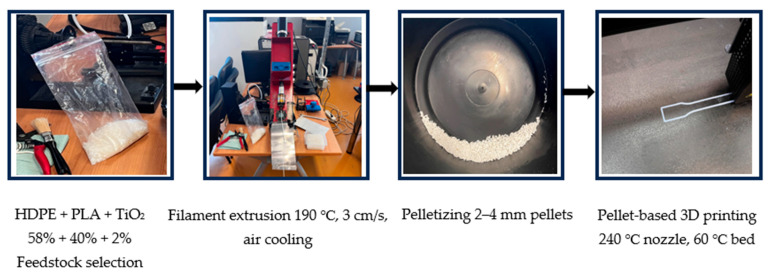
Experimental workflow used for the development of the HDPE–PLA–TiO_2_ composite, including feedstock selection, filament extrusion, pellet preparation, and pellet-based 3D printing.

This stepwise sequence, presented in Scheme 1, is consistent with recent methodological frameworks proposed for recycling-oriented additive manufacturing, in which material preparation, extrusion or re-extrusion, digital model preparation, printing, and final mechanical evaluation are treated as interdependent stages of the same manufacturing chain [11].

### 2.3. Filament Extrusion

The composite was first processed using a Noztek Pro filament extruder (Noztek, Shoreham-by-Sea, UK). Extrusion was performed at a processing temperature of 190 °C, with a screw advance speed of 3 cm/s. After exiting the die, the material was cooled using an air flow at approximately 15 °C. Under these processing conditions, the addition of PLA contributed to improved dimensional stability of the extrudate and reduced the tendency of HDPE to expand after exiting the nozzle. The extruded material was obtained as a filament with an average diameter of approximately 3 mm, corresponding to the selected nozzle size.

After extrusion, the filament was cut into pellets with lengths between 2 and 4 mm in order to allow subsequent processing in a pellet-fed additive manufacturing system.

### 2.4. Manufacturing of Test Samples

The pellets obtained after grinding were used to produce the specimens using a PioCreat G5 Pro 3D printer (Piocreat, Shenzhen, China). Preliminary tests were carried out in order to identify the optimal processing conditions for their fabrication. Based on these tests, the following optimal fabrication parameters were established: nozzle temperature of 240 °C, printing bed temperature of 60 °C, layer height and initial layer height of 0.4 mm, line width and top/bottom layer thickness of 0.8 mm, printing speed of 40 mm/s, wall printing speed of 50 mm/s, a single outer wall with a thickness of 1 mm, and a balanced zig-zag infill pattern.

A zig-zag infill strategy was selected to ensure a uniform deposition of the material. A constant distance between the nozzle and the printing bed was maintained for all printed specimens used for tensile testing. The same parameters were kept in order to reduce warping, dimensional instability, poor adhesion during printing, and the formation of voids within the matrix.

### 2.5. Thermal Characterization

The thermal characterization was carried out using a simultaneous thermal analyzer STA 449 F3 Jupiter (Netzsch, Selb, Germany). Thermogravimetric analysis (TG) and differential thermal analysis (DTA) were performed in order to evaluate the thermal stability of the created product, the degradation profile and the thermal transitions that occur in the material, as well as the compatibility of the material with the temperatures selected for extrusion and printing.

The measurements were performed in the temperature range of 0–450 °C, with a gradual heating rate of approximately 50 °C/min, for samples of approximately 10 mg. A total of 5 samples were tested.

### 2.6. Mechanical Characterization

The mechanical tests were carried out using a universal testing device WDW-150S (Jinan Testing Equipment Corporation, Jinan, China) and Matest S.p.A (Treviolo (BG), Italy), in accordance with the requirements of ISO 7500-1 [38] and ISO 376 [39], respectively. In the first part, testing was performed on randomly selected filament-type specimens taken from the extruded filament, in order to obtain the first mechanical results of the material. In the second stage, the composite specimens were printed and subsequently tested in order to determine the tensile strength, later comparing the obtained values.

For the tests, the SR EN ISO 2062 standard [40] was used in the case of the filaments, with a crosshead speed of 50 mm/min, and for the printed samples, we used ISO 527 [41], with a crosshead speed of 5 mm/min. The specimens were mounted in the tensile grips of the testing machine and carefully aligned along the loading axis. They were evaluated comparatively based on the tensile response and post-test visual observation. A systematic classification of fracture modes and a percentage-based quantification of their occurrence for each printing condition were not included in the paper, remaining subjects for future investigation.

### 2.7. Morphological and Compositional Characterization

**Figure 1:**
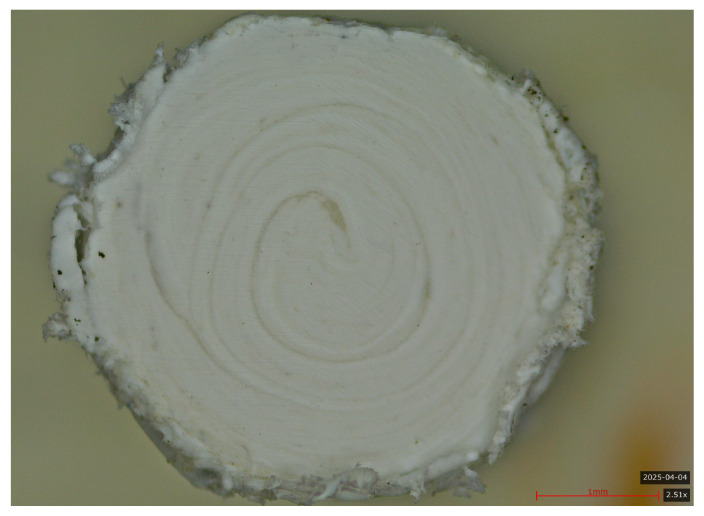
Optical microscopy image showing the cross-section of the extruded HDPE–PLA–TiO_2_ composite filament.

Using an optical microscope with interchangeable lenses, Leica Emspira 3 model, morphological observations were performed. The investigated surface corresponds to the extruded filament after grinding, as illustrated in Figure 1.

SEM coupled with EDS was used for a preliminary investigation of the fracture surfaces, as well as the local elemental distribution of components in the printed composite. The analysis was achieved using a Tescan Vega LMU scanning electron microscope, operated in high-vacuum mode. SEM images were obtained at an accelerating voltage of 20 kV, while EDS analysis was performed using a beam current of 300 pA. Secondary electron (SE) images were used for the evaluation of the surface morphology of the fractured samples. The images obtained through compositional contrast electrons showed the local variations, which may be associated with compositional or topographical differences. EDS elemental mapping was performed for carbon, oxygen, and titanium, confirming the presence of particles containing Ti and the qualitative evaluation of their distribution in the polymer matrix. Due to the roughness and the irregular geometry of the fracture surfaces, the maps were considered qualitative and were not used for the quantitative determination of nanoparticle dispersion.

## 3. Results

### 3.1. Development of the Composite and Printability Improvement

**Figure 2:**
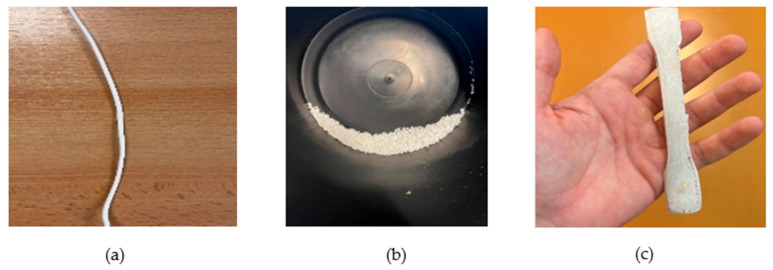
Composite processing and preliminary printing behavior: (**a**) composite filament extrusion; (**b**) pelletized HDPE-PLA-TiO_2_ material; (**c**) preliminary tensile sample obtained before optimization.

The idea of developing the HDPE–PLA–TiO_2_ component system started from the identification of a major limitation in the processing of HDPE during the extrusion stage, namely the pronounced volumetric increase, possibly caused by the humidity in the room, at the exit from the nozzle. This aspect led to problems in the filament geometry through non-uniformity, as well as to difficulties in obtaining a reproducible material for the subsequent manufacturing stages. The main processing stages are shown in Figure 2. Figure 2a illustrates the composite filament extrusion, Figure 2b shows the pelletized HDPE–PLA–TiO_2_ material, and Figure 2c presents the preliminary tensile sample obtained before optimization.

In this composite, PLA acts as a secondary polymeric phase that contributes to improved melt processability and limits the uncontrolled expansion observed during the extrusion of HDPE-rich formulations. The improvement in filament regularity and printability should not be interpreted as proof of complete compatibility between HDPE and PLA or as quantitative evidence of uniform TiO_2_ dispersion, but rather as an indication of improved macroscopic processing stability under the selected extrusion conditions. Under the conditions in which extrusion was carried out according to Section 2.3, the obtained filaments showed superior reproducibility compared with the initial HDPE-rich ones. The micrograph in Figure 1 indicates a relatively continuous morphology of the extruded filament at the optical scale.

This intermediate stage demonstrates that the formulation was sufficiently stable not only for the extrusion process in the form of a filament, but also for reprocessing in the form of pellets without destroying the structure.

At the beginning of the first printing tests, in which only HDPE and TiO_2_ were used, non-homogeneous specimens with visible voids were obtained. This aspect confirms that the initial system did not provide satisfactory printing behavior; for this reason, PLA was introduced into the composite.

**Figure 3:**
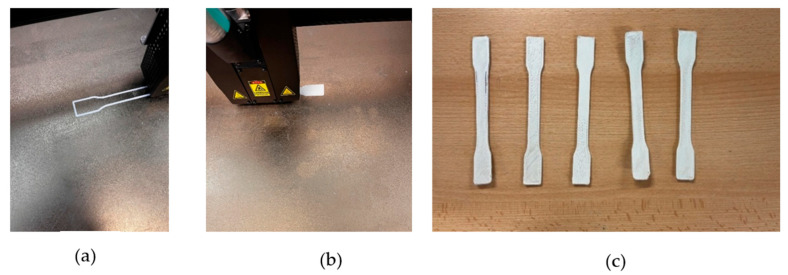
Pellet-based 3D printing and specimen fabrication; (**a**) test sample construction; (**b**) material filling during printing; (**c**) constructive evolution/final printed tensile specimens.

By introducing PLA and applying the optimized processing conditions detailed in Section 2.4, the fabrication of test specimens became possible, resulting in compact and stable parts. The pellet-based printing process and specimen fabrication are shown in Figure 3. Figure 3a illustrates the construction of the test sample, Figure 3b shows the material filling stage, and Figure 3c presents the final printed tensile specimens. These images demonstrate that, after the introduction of PLA and the use of the optimized processing conditions, compact and stable tensile specimens could be fabricated. The improvement observed compared with the preliminary HDPE–TiO_2_ specimen shown in Figure 2c is attributed to the compositional modification and improved processing stability.

### 3.2. Thermal Behavior of the HDPE–PLA–TiO_2_ Composite

**Figure 4:**
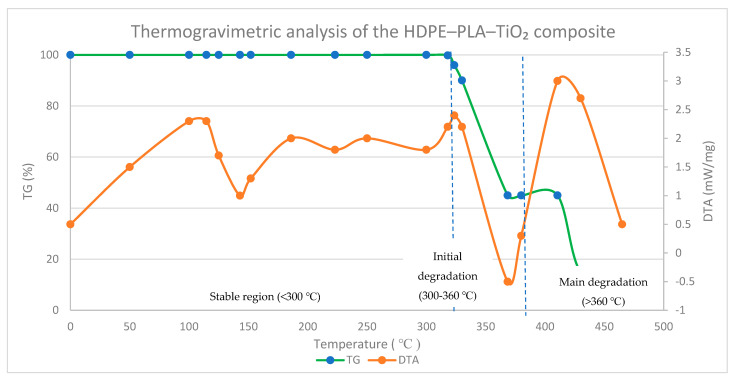
Thermogravimetric analysis of the HDPE–PLA–TiO_2_ composite.

The results obtained according to Figure 4 show the thermal behavior of the composite analyzed simultaneously by TG/DTA, in order to assess how stable it is during processing. The obtained curves indicate a stepwise thermal response, characteristic of a heterogeneous polymeric system.

The TG curve showed that the sample mass remained essentially unchanged up to approximately 300 °C, with a minor variation of 0.68%. This result indicates that no major mass-loss degradation occurred within the temperature range used for extrusion and pellet-fed 3D printing under the applied TG/DTA conditions. Therefore, the selected processing temperatures were below the main degradation stage detectable by thermogravimetric mass loss.

Although the TG curve shows only a minor mass variation below approximately 300 °C, this observation must be interpreted with caution. TG/DTA provides information mainly on mass-loss behavior and thermal events under dynamic heating conditions, but it cannot demonstrate the absence of molecular degradation, molecular weight reduction, viscosity changes, or crystallinity modifications. Therefore, the absence of significant mass loss below approximately 300 °C indicates that no major volatile degradation products were detected under the applied testing conditions, but it does not exclude possible molecular-level degradation of the PLA phase during extrusion or pellet-fed printing.

The actual thermal exposure during printing depends not only on nozzle temperature, but also on residence time, melt flow, heat transfer, screw feeding, and the HDPE-rich composition of the blend. Consequently, the processing temperature of 240 °C should be considered acceptable only in relation to the observed macroscopic processing behavior and TG/DTA mass-loss response, while further differential scanning calorimetry (DSC), rheological, melt flow index, and molecular weight investigations are required to fully assess the thermal history and degradation mechanisms of the PLA-containing system.

The DTA curve highlights a first thermal process at 114.6 °C, followed by a more pronounced transformation at 142.9 °C. These effects may mean melting or structural rearrangements of the polymer phases inside the composite. The presence of several state changes in the system is nevertheless understandable due to its nature, as it is a structure with distinct thermal contributions from HDPE and PLA, but with influences from the TiO_2_ particles. At approximately 323.5 °C, an additional transformation is observed, showing the onset of new structural changes before the decomposition process. Around 368.5 °C, the most significant transformation took place, showing a mass loss of approximately 52%, clearly signaling the degradation of the organic fractions in the composite. The decomposition process continues progressively up to approximately 490 °C, showing a stepwise degradation of the polymer mass. At the end of the TG run, the maximum cumulative mass loss of the HDPE–PLA–TiO_2_ composite was estimated from the final residual mass of the TG curve. The composite showed a maximum mass loss of approximately 85–90%, leaving about 10–15% residual mass, which can be mainly associated with the inorganic TiO_2_ phase and possible carbonaceous residues. In thermogravimetric analysis, two main degradation stages are commonly reported, with the lower-temperature stage being predominantly associated with PLA chain scission, while the higher-temperature stage is attributed to the degradation of the HDPE matrix [33,34]. In the composite, the mass-loss interval beginning approximately between 300 and 360 °C can therefore be associated mainly with PLA, whereas the more intense degradation above 360 °C is attributed mainly to HDPE decomposition. The TiO_2_ phase is thermally stable within this interval and is associated with the residual inorganic fraction.

### 3.3. Tensile Performance of the Extruded Filaments

The mechanical results of the extruded filaments shown in Table 1, were evaluated by tensile tests carried out on randomly selected segments. The results showed a relatively fine strength interval, varying between 20 and 25 MPa. The elastic modulus increased, reaching approximately 2 GPa, a value that exceeds regular HDPE, but which is justified due to the introduction of PLA, which is more rigid, while TiO_2_ particles also appear and strengthen the rigidity.

**Figure 5:**
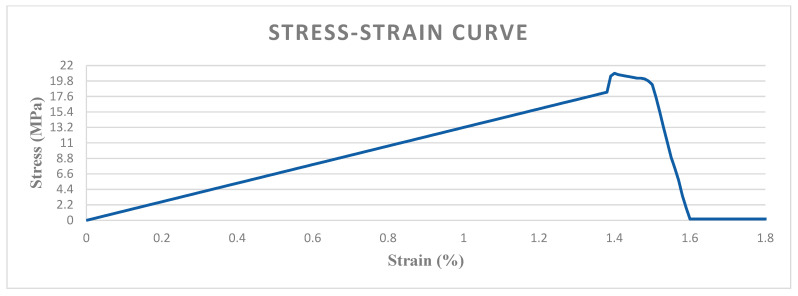
Tensile test of sample 2 (T2).

**Figure 6:**
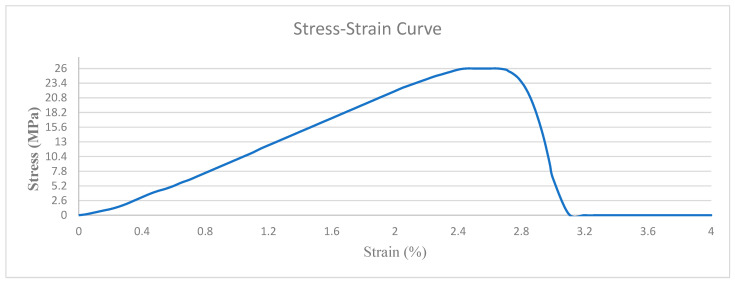
Tensile test of sample 6 (T6).

The tensile performance is satisfactory; however, the results still highlighted problems of local inhomogeneity. Some of these were more brittle, which shows that local TiO_2_ agglomerations could exist, due to imperfect dispersion during formation. Samples T3 and T6 showed a more favorable behavior, showing a maximum tensile strength and clear delimitations between the yield points. The corresponding data are summarized in Table 2 and Table 3 and graphically are presented in Figure 5 and Figure 6. The results thus determine that, although the composite tends to increase its rigidity, it still presents a degree of ductility.

In Table 2 and Table 3, S0 denotes the initial cross-sectional area, L0 the initial gauge length, Le the effective test length, Rn the tensile strength, Rp the yield strength, Rt the stress at break, Fm the maximum force, Fp the force at the yield point, Ft the breaking force, E the Young’s modulus, and At the elongation at break.

### 3.4. Thermal Stability of the 3D-Printed Test Samples

**Figure 7:**
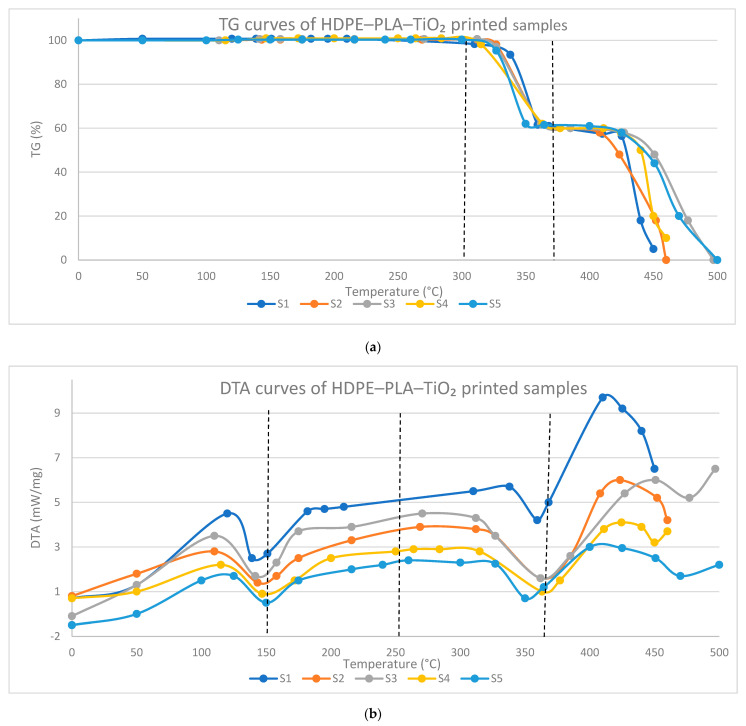
Thermal behavior of the HDPE–PLA–TiO_2_ 3D-printed specimens: (**a**) TG curves and (**b**) DTA curves.

The evaluation of the thermal stability of the 3D-printed specimens was carried out by TG/DTA in order to determine whether the additional thermal history associated with pellet-based fabrication produced major changes detectable through mass-loss analysis. Figure 7a shows the TG curves of the printed specimens, while Figure 7b presents the corresponding DTA curves. These profiles suggest that the second thermal cycle associated with pellet-based printing did not produce major changes detectable by TG/DTA. However, this result should not be interpreted as proof of the absence of molecular degradation.

The plots show similar thermal behavior among the printed samples, with mass stability below approximately 300 °C, followed by progressive degradation at higher temperatures.

The TG curves showed that all tested samples remained stable in terms of mass loss up to approximately 300 °C, with mass losses below 1%. This behavior indicates that no major mass-loss degradation occurred within this temperature range under the applied conditions. Instead, the small mass variations can be mainly associated with the removal of residual moisture, rather than with substantial decomposition of the polymeric structure.

The results should not be interpreted as evidence that molecular degradation was completely absent.

The first observable degradation stage is between the temperatures of 300 °C and 360 °C, where the samples lose approximately 5 to 7% of their mass. In this temperature range, the onset of the degradation of the polymer chains appears, and above the temperature of 360 °C, there is a mass loss of between 34 and 37%, representing the main stage of decomposition and degradation of the material. Complete decomposition is reached at an approximate value of 430 °C for all the investigated samples.

The DTA curves additionally confirm the thermal consistency of the printed materials. A first endothermic event appears at approximately 150 °C in all specimens. The second thermal event appears around a value of 250 °C, which is associated with a partial recrystallization or structural rearrangement within the system. Up to the temperature of 360 °C, no considerable changes appear, where the main degradation stage begins, which is also observable on the TG curve and influencing the breaking of the chains in the matrix. At the end, close to the temperature of 430 °C, the complete decomposition and the presence of residues are marked.

### 3.5. Tensile Performance of the 3D-Printed Samples

**Figure 8:**
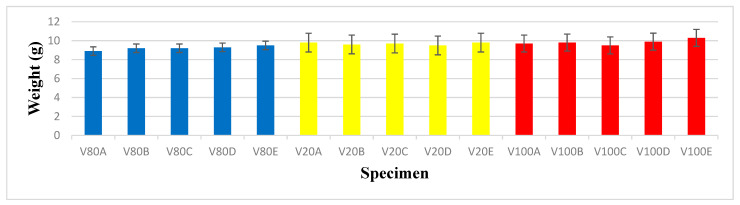
Variation in Specimen Weight.

The mechanical results of the specimens were evaluated accordingly using three groups of samples. These were designated V20, V80, and V100; this distinction does not indicate different compositions, but rather different printing conditions applied to the same composite. Their printing was carried out while maintaining the nozzle and bed temperatures constant, whereas the printing speed and feed rate were varied in order to identify the most favorable parameter set. Five specimens were produced for each group under the same conditions; however, a variation in weight between 8.9 g and 10.3 g was observed, as shown in Figure 8.

The variation in weight is attributed to the intrinsic dosing fluctuations specific to pellet-fed deposition, slight differences in the overlap of the deposited material layers, local void content, and the start–stop printing behavior of the screw-based feeding system.

**Figure 9:**
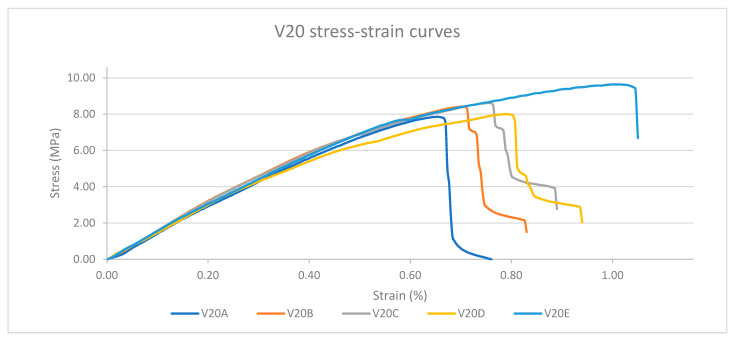
Representative stress–strain curves for the V20 group.

**Figure 10:**
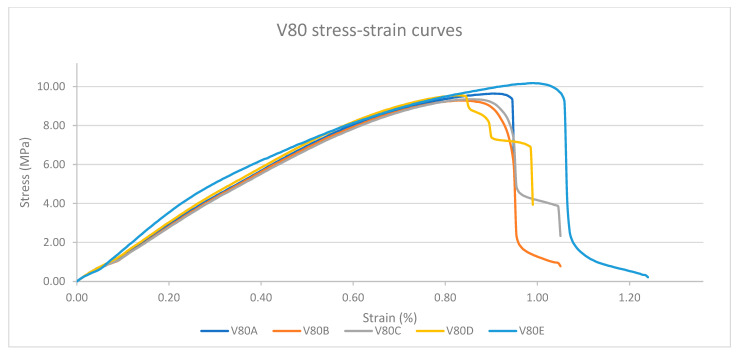
Representative stress–strain curves for the V80 group.

**Figure 11:**
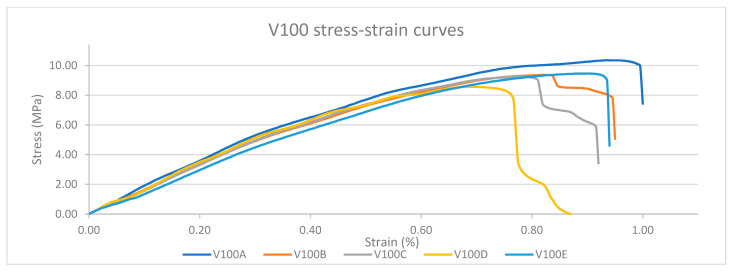
Representative stress–strain curves for the V100 group.

The V80 group exhibited the highest average tensile strength, approximately 9.60 MPa, followed by V100 with approximately 9.40 MPa, while the V20 group showed the lowest average value, approximately 8.51 MPa. The average strain at break was approximately 1.06% for V80, 0.94% for V100, and 0.89% for V20. In terms of strain at break, specimen V80E recorded the highest value, reaching 1.24%. The representative stress–strain curves corresponding to the three investigated printing conditions are presented in Figure 9, Figure 10 and Figure 11. Figure 9 shows the tensile response of the V20 group, Figure 10 presents the behavior of the V80 group, and Figure 11 illustrates the stress–strain curves obtained for the V100 group.

Compared with the force–elongation curves, the stress–strain curves provide a more representative comparison of the tensile behavior because they normalize the response with respect to specimen geometry. While the force–elongation plots illustrate the overall load-bearing capacity and extension up to failure, the stress–strain representation better highlights the intrinsic tensile response of each processing condition. In this respect, the V80 group showed the most balanced mechanical behavior, combining the highest average tensile strength, the highest average strain at break, and the lowest data dispersion among the investigated groups.

As shown in Figure 9, Figure 10 and Figure 11, all printed specimens exhibited a similar initial tensile response, followed by a sudden stress drop associated with fracture. Among the investigated groups, the V80 specimens showed the most balanced behavior, with relatively high tensile strength and improved deformation before failure.

**Figure 12:**
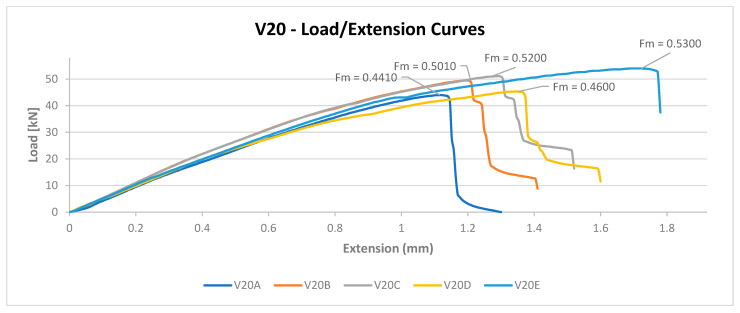
Representative tensile load–elongation curves for the V20 group.

**Figure 13:**
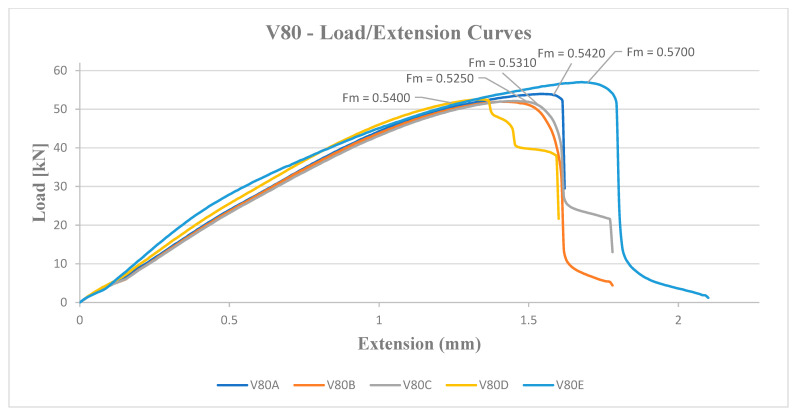
Representative tensile load–elongation curves for the V80 group.

**Figure 14:**
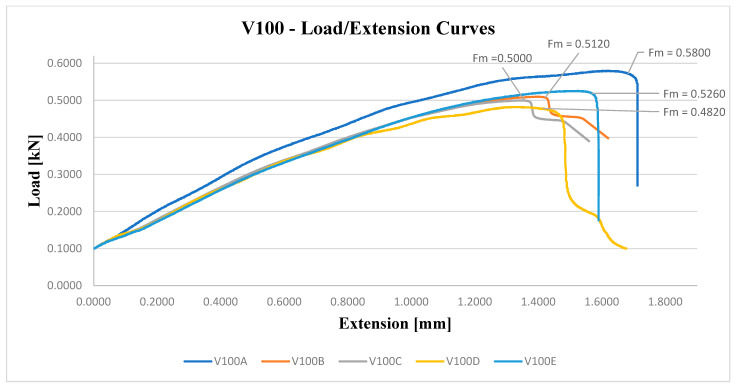
Representative tensile load–elongation curves for the V100 group.

The tensile strength results, as shown in Table 4, were between 7.86 MPa and 10.36 MPa, and the elongation at break was between 1.7 mm and 2.1 mm. The corresponding load–elongation curves are presented in Figure 12, Figure 13 and Figure 14. Figure 12 shows the V20 group, Figure 13 presents the V80 group, and Figure 14 illustrates the V100 group. These curves provide complementary information regarding the load-bearing capacity and elongation behavior of the printed specimens. According to the data in Table 4, the highest elongation at break within the V80 group was recorded for specimen V80E (2.1 mm). This behavior is consistent with a slightly higher deposited mass and a likely better local interlayer continuity, which delayed crack initiation and allowed a marginally larger deformation before failure.

The comparison between Figure 12, Figure 13 and Figure 14 confirms that the V80 printing condition provided the most favorable combination of load-bearing response and elongation before fracture, while the V20 and V100 groups showed slightly lower or more variable mechanical behavior.

### 3.6. Statistical Analysis of the Mechanical Results

**Figure 15:**
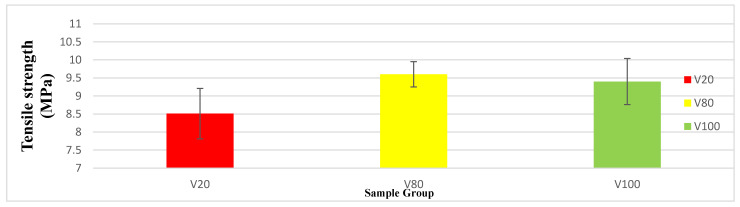
Average tensile strength of HDPE–PLA–TiO2 printed specimens obtained under different printing conditions.

In order to determine whether the printing conditions had a significant role, from a statistical point of view, on the tensile tests, an analysis of variance (ANOVA) was carried out using the tensile strength values of the three groups from Table 4 and Figure 15.

The bars represent the experimental mean values, while the error bars indicate the standard deviation corresponding to each tested group of samples (*n* = 5). Different letters associated with the bars indicate the presence of statistically significant differences among the three analyzed groups. In the statistical study, groups with printing values of 60% speed/80% feed (V80), 20% speed/100% feed (V20), and 100% speed/100% feed (V100) were used, with five replicates for each group shown in Table 5.

The standard deviation values further emphasize the reproducibility of the process, with the V80 group exhibiting the lowest data dispersion and, consequently, the most consistent tensile behavior. In contrast, the greater dispersion observed for the other two groups, V20 and V100, suggests a less homogeneous structural response, apparently due to differences in interlayer adhesion during material deposition.

The statistical analysis shows that the V80 group has the highest tensile strength values, having a mean of 9.6 MPa and the smallest dispersion, SD = 0.353 MPa, giving this group increased reproducibility. At the opposite pole, the V20 group showed the lowest values, having a mean of 8.51 MPa and the largest dispersion, SD = 0.703 MPa. The last group, V100, is close in mean value to V80, having a mean tensile strength of 9.40 MPa, but the deviation is approximately twice as high, SD = 0.640 MPa.

### 3.7. SEM–EDS Morphological and Elemental Analysis

**Figure 16:**
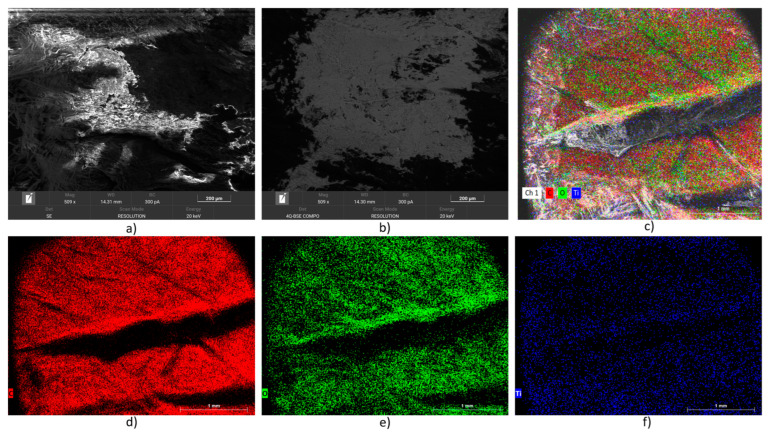
SEM–EDS analysis of the fracture surface of the printed HDPE–PLA–TiO_2_ composite: (**a**) SEM-SE micrograph; (**b**) BSE/COMPO image; (**c**) combined C/O/Ti elemental map; (**d**) C-K elemental map; (**e**) O-K elemental map; (**f**) Ti-Kα elemental map.

The SEM–EDS analysis shows the relationship between the morphology of the fractured surfaces and the mechanical behavior of the composite, shown in Figure 16. The preliminary microstructural investigations highlight cavities, microcracks, and the fibrillar structure of the material. The images obtained through compositional contrast electrons showed the local variations, which may be associated with compositional or topographical differences. The developed cavities, microcracks, and a heterogeneous fracture surface, are characteristic of a non-uniform fracture mechanism involving localized plastic deformation. The presence of cavities and discontinuities focusses on some stress concentration areas, which may reduce the tensile strength observed in the printed specimens.

The Backscattered Electrons/compositional contrast (BSE/COMPO) images show up contrast variations within the fractured surface. The EDS maps confirm the presence of C, O, as expected, due to the nature and starting composition of the materials and Ti in the analyzed region. Carbon is associated with the polymeric matrix, while oxygen may belong both to the PLA phase and to the TiO_2_ particles. The Ti map shows the presence of titanium-containing particles within the composite, supporting the successful incorporation of TiO_2_ into the HDPE–PLA matrix.

The Ti-containing regions identified in the analyzed fractured area indicate that the TiO_2_ distribution is not fully homogeneous, since local regions with higher Ti signal intensity can be observed. These areas may be associated with TiO_2_-rich domains or particle agglomerations. These agglomerations, together with the cavities and microcracks observed in the SEM images, may partially explain the moderate tensile strength of the specimens. Therefore, the SEM–EDS results support the idea that the mechanical behavior of the composite is controlled not only by the formulation, but also by the local morphology generated during pellet-based deposition.

Since the EDS maps were acquired on rough fractured surfaces, the results should be interpreted as qualitative evidence of TiO_2_ incorporation and distribution within the material, rather than as a fully quantitative evaluation of nanoparticle dispersion.

## 4. Discussion

### 4.1. Effect of PLA on Printability and Extrusion Stability

This progressive improvement demonstrates that the optimization of the formulation and of the processing parameters was essential in transforming a difficult-to-print HDPE-rich system into a material suitable for pellet-fed manufacturing. However, HDPE and PLA are generally considered poorly compatible polymers due to their different polarities and molecular structures. Since no coupling agent or compatibilizer was used in the present formulation, the improved printability observed in this study should not be attributed to complete interfacial compatibility between the two phases. Instead, PLA should be regarded as a secondary polymeric phase that contributed to improved processing stability, reduced uncontrolled expansion during extrusion, and increased rigidity of the final composite. The observed improvement in filament regularity and pellet-fed printability therefore reflects a processing advantage of the selected formulation rather than a complete morphological homogenization of the HDPE–PLA system.

For clear highlighting, a comparison was made. First of all, we have the reference point of neat HDPE, which usually presents tensile strength values of 30–35 MPa, while its composites show values of approximately 20 MPa, depending on the processing conditions [7,8]. In this study, the extruded filament showed tensile strengths of 20–25 MPa, providing competitive performance compared with other HDPE-based systems presented in the literature. PLA and TiO_2_ allowed the development of a stiffer and easier-to-process composite, which has suitable properties for manufacturing and later for transformation into pellets.

The tensile results obtained from the tests confirm the presence of a useful balance between rigidity and processability. This is a promising result from the perspective of applicability, because the material through extrusion must present adequate strength and maintain appropriate structural stability during its processing.

### 4.2. Thermal Stability and Suitability for Melt Processing

The thermal behavior observed in the TG/DTA supports the suitability of the material for the selected extrusion and pellet-fed printing conditions from the point of view of mass-loss stability. Indeed, the presence of 40 wt.% PLA requires a cautious interpretation, due to the nature of PLA, which may undergo molecular-level degradation during melt processing, even before major mass loss becomes visible in TG. The discussion regarding thermal stability in this study mainly refers to the absence of significant mass loss and to the preservation of a comparable TG/DTA profile after processing, not to the complete absence of chain scission, molecular weight reduction, or changes in the crystalline structure of the composite.

### 4.3. Mechanical Behavior of the Printed Specimens

The results confirm that the material can be processed in the form of self-supporting printed parts with moderate mechanical strength. Although the tensile strength values of the printed specimens are moderate, they should be interpreted in relation to the pellet-based deposition process and the HDPE-rich multiphase formulation. The printed specimens showed tensile strength values between 7.86 and 10.36 MPa, whereas the extruded filaments reached 20–25 MPa. This reduction is mainly attributed to the layered structure of the printed parts, limited interlayer adhesion, local void formation, deposition non-uniformity, and feeding fluctuations specific to screw-based pellet deposition. Moreover, the HDPE–PLA–TiO_2_ system was processed without a coupling agent or compatibilizer; therefore, the material should not be interpreted as a fully compatibilized polymer blend. The mechanical performance of the printed samples should not be interpreted only as an intrinsic property of the composite formulation, but also as the result of the interaction between material composition and printing conditions.

The differences between the V20, V80, and V100 groups indicate that the deposition regime strongly influenced the final tensile response. Since all groups were produced from the same HDPE–PLA–TiO_2_ composition, the observed differences are attributed to changes in printing speed and feed rate, which can affect material flow, local cooling, layer overlap, void formation, and interlayer consolidation. In this context, the V80 condition provided the most balanced mechanical response, combining the highest average tensile strength with the lowest data dispersion.

Among the three investigated groups, the best tensile response was that of those whose values are close to 10 MPa, while the group that remained below 8 MPa was rated as the weakest, mainly due to the different process parameters used during manufacturing which significantly affect these features. The variation between values shows us that the mechanical performance of the printed parts is sensitive to even the smallest modifications such as printing speed or feeding conditions. The structural integrity after printing demonstrated by the results suggests the stability of the deposition regime, rather than major differences in the intrinsic behavior of the material.

This sensitivity to processing conditions is in agreement with recent FGF studies showing that nozzle temperature, layer height, deposition orientation, and porosity control the final tensile response of printed composites [33,34]. In the present case, the V80 condition provided the best balance between deposition stability and material supply, while the V20 condition likely promoted less favorable interlayer consolidation.

**Figure 17:**
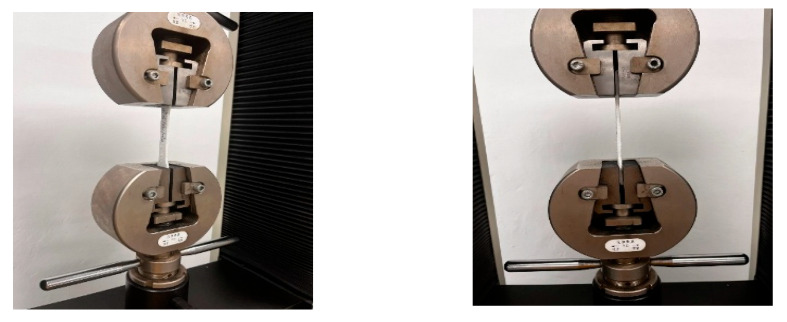
Test sample subjected to tensile testing.

The elongation at break is relatively low, indicating that the printed material presents a rigid response, a behavior in agreement with the increase in rigidity observed previously both at the level of the filaments and of the specimens. PLA contributes to the increase in rigidity, TiO_2_ additionally limits the mobility of the polymer chains in the matrix, thus resulting in a less deformable structure. Figure 17 shows the tensile testing configuration used for the mechanical evaluation of the printed samples.

The tensile results of the printed specimens indicate a system with a functional balance between printability and mechanical integrity. The developed composite was not intended to surpass neat PLA in terms of absolute tensile strength; instead, it was designed to improve the printability of an HDPE-rich system while maintaining a functional balance between processability, thermal stability, and mechanical integrity under pellet-based manufacturing conditions. The mechanical strength values remain below those of the filaments, which can be explained by the positioning of the printable layers and possible deposition non-uniformities. Through these findings, the path for future improvements is already established, which should focus on the homogeneity of deposition and on the quality of the bonds between layers. The material itself showed adequate thermal stability and promising properties during the verification of the filaments.

### 4.4. Statistical Significance and Overall Practical Relevance

The ANOVA results present a statistically significant effect of the printing conditions on strength, with F (2,12) = 4.93 and *p* = 0.027. The results show that the variation in the deposition regime was sufficient to produce a measurable difference in the mechanical performance of the parts. The poor performance of the V20 group shows that reducing the printing speed to 20% simultaneously with increasing the feed rate to 100% does not improve the adhesion of the deposited structure, but increases the exposure to cooling and at the same time reduces the mean tensile strength through the lack of interlayer adhesion. In the case of the V80, it produced the most stable and balanced parts in terms of mechanical response out of the three categories, and V100 led to acceptable strengths but to a reproducibility inferior to the V80 group.

The statistically significant difference was observed between the V20 and V80 groups, having *p* = 0.030, while the differences between V20 and V100 had *p* = 0.08, and V80 and V100 had *p* = 0.848. These results suggest that the mechanical penalty associated with the V20 group is sufficiently pronounced to differentiate the most balanced processing regime. The statistical analysis confirms the reproducibility obtained under the V80 printing conditions for this composite, offering a good balance between strength and tensile behavior.

The statistical analysis confirms that the printing conditions had a measurable influence on the tensile performance of the printed specimens. The V80 group showed the highest average tensile strength, approximately 9.60 MPa, and the lowest standard deviation, SD = 0.353 MPa, indicating the most reproducible behavior among the tested conditions. In contrast, the V20 group showed the lowest average tensile strength, approximately 8.51 MPa, and the highest dispersion, SD = 0.703 MPa, suggesting less stable deposition and weaker interlayer consolidation. The V100 group reached an average tensile strength close to that of V80, approximately 9.40 MPa, but with a higher standard deviation, SD = 0.640 MPa, indicating reduced reproducibility.

These results support the interpretation that the developed material is sensitive to the deposition regime, which is typical for pellet-fed additive manufacturing of multiphase polymer systems. The mechanical results should therefore be interpreted as evidence of a processability-optimized composite with moderate tensile performance and increased rigidity, rather than as a material designed to outperform conventional PLA or neat HDPE in terms of absolute tensile strength.

The statistical evaluation of the specimens demonstrates that the properties are significantly influenced by the processing parameters selected during production. Consequently, the improvement of deposition stability and cohesion between layers should represent a main direction of future optimization, because the current formulation provides the appropriate basis for manufacturing, conferring thermal stability and mechanical viability.

The overall results obtained in the previous subchapters indicate that a favorable balance was achieved between the processability, thermal stability, and mechanical performance of the proposed material. By introducing PLA into the HDPE matrix, the extrusion stability is significantly improved, which allows for the obtaining of uniform filaments and pellets, while the introduction of TiO_2_ adds additional functional value through its stabilizing role. The final composite does not show behavioral signs of an HDPE-rich system that would show processing difficulties, but rather of a stable multiphase system, compatible with 3D printing.

From a thermal point of view, it presents the best general behavior among the compared materials, with a characteristic transformation around 150 °C and the appearance of degradation close to 360 °C, which corresponds to thermal stability. In comparison with the reference systems and even the recycled ones, it presents higher characteristic temperatures and fewer secondary thermal transformations, further strengthening the stability and a well-defined structural evolution. The results are particularly relevant for repeated processing in the melting of the material, confirming that the temperatures selected for extrusion and printing remain below the degradation range.

Through the mechanical results, supporting elements of the material are also obtained in turn. Neat HDPE generally shows higher tensile strength, but through the introduction of PLA and TiO_2_ the rigidity is increased, and the mechanical values are maintained within a competitive range for polymer composites. The filament demonstrated satisfactory values in the tensile tests, unlike the printed parts, in which an expected reduction in strength is observed because of interlayer effects and the local non-uniformity of the layer deposits. Despite all these difficulties, the specimens preserved sufficient structural integrity for applications in which their stability, moderate rigidity, and thermal resistance are more important than the elongation capacity.

An important result of the present investigation is present in the final properties of the composite, which are modified by the deposition conditions during printing. The data show that by varying the feed rate and the printing speed, the uniformity and homogeneity of the printed parts are influenced. In turn, they generate differences in the mechanical measurements, an aspect that shows that processing optimization is just as important as the design of the material itself.

All the same time, the mechanical and thermal results highlight practical advantages related to processing and reuse. The importance of this aspect can be found in large-scale manufacturing and in the development of sustainable materials, especially for recycled or partially recycled components.

Overall, the HDPE–PLA–TiO_2_ composite groups together a set of advantageous characteristics through improved printability compared with classic HDPE, increased rigidity, and acceptable strength of the printed parts. This set of characteristics makes the proposed material promising for additive manufacturing applications, where a balanced combination between processability and performance in service is desired. In the future, the improvements that should appear are concentrated on strengthening between layers, reducing local heterogeneity, and refining the processing parameters in order to obtain an increase in the reproducibility of the printed components.

### 4.5. Limitations of the Study and Future Research Directions

The scientific contribution of this study lies in the experimental demonstration and critical evaluation of a complete processing route for an HDPE-rich multiphase composite, including extrusion, pellet preparation, pellet-fed 3D printing, and thermo-mechanical assessment. This approach provides practical and comparative information for the development of pellet-printable polymer blends and identifies the main processing and characterization aspects that require further investigation.

The present study focused on the feasibility of processing an HDPE-rich formulation through extrusion, pelletizing, and pellet-fed 3D printing, with emphasis on processability, thermal mass-loss behavior, and tensile performance. The SEM–EDS observations provide some preliminary results on the material obtained and valid morphological support for the mechanical results. The printed specimens showed lower tensile strength than the extruded filaments, which can be related to the combined effect of pellet-based deposition, interlayer adhesion limitations, local void formation, and heterogeneous fracture morphology. The presence of cavities, microcracks, fibrillar structures, and material pull-out indicates that failure occurred through a non-uniform mechanism. In addition, the EDS maps confirmed the presence of Ti-containing particles within the polymer matrix, while local Ti-rich regions can be associated with possible TiO_2_ agglomeration. These morphological and compositional heterogeneities may act as stress concentration sites, reducing effective stress transfer, and contribute to the results concerning the moderate tensile performance of the printed specimens.

Similarly, the TG/DTA results demonstrate the absence of significant mass loss under the applied dynamic heating conditions, but they do not exclude possible molecular-level degradation of PLA during extrusion or pellet-fed printing. Therefore, future work should include DSC, rheological analysis, MFI measurements, and molecular weight evaluation in order to assess possible changes in crystallinity, viscosity, chain scission, and molecular structure after processing.

The mechanical characterization was mainly based on tensile testing of printed specimens obtained under selected processing conditions, with five specimens tested for each group and statistical analysis applied. Additional investigations, including flexural testing, impact behavior, fracture surface analysis, fatigue behavior, and long-term durability, are necessary in future studies for a more complete evaluation of the material.

## 5. Conclusions

The present study demonstrated the feasibility of developing an HDPE–PLA–TiO_2_ composite for pellet-based additive manufacturing through 3D printing. The addition of PLA improved extrusion stability and reduced the uncontrolled expansion usually associated with HDPE-rich formulations, allowing the production of filaments, pellets, and printed specimens. TiO_2_ was incorporated as a filler with a potential contribution to rigidity, residual thermal fraction, and UV-screening functionality.

TG/DTA did not show any significant mass loss at temperatures below approximately 300 °C under the applied testing conditions, indicating behavior suitable for extrusion and printing. However, the results obtained from this analysis should not be interpreted as proof of complete molecular stability, and additional DSC, rheological, and related analyses are necessary to evaluate the possible degradation of PLA during processing.

The study showed that the extruded filaments provided tensile strength values of 20–25 MPa, while the printed samples exhibited tensile strengths between 7.86 and 10.36 MPa. The V80 group showed the best overall tensile response, with an average tensile strength of approximately 9.60 MPa and the lowest standard deviation. This confirms that the final mechanical behavior was strongly influenced by the deposition regime and not only by the composite formulation.

Some preliminary SEM–EDS analysis confirmed the presence of Ti-containing particles within the printed composite and revealed a heterogeneous fracture morphology with cavities, microcracks, fibrillar structures, and local Ti-rich regions. These observations sustain the assumption that the moderate tensile strength of the printed specimens is related to the combined influence of pellet-based deposition, local void formation, heterogeneous fracture morphology, and possible TiO_2_ agglomeration.

The proposed HDPE–PLA–TiO_2_ composite has the potential to be considered a processability-optimized multiphase material, rather than a fully compatibilized or completely microstructurally validated composite. The results provide strong points for the further improvement and development of the material and support its potential as a material suitable for rigid or semi-structural pellet-printed components.

## Figures and Tables

**Table 1:** Results of the tensile testing of the filaments.

Wire Specimen	Initial Length	Final Length	Diameter	Tensile Strength	Yield Strength (Rp)	Modulus of Elasticity (E)	Error Values
-	[mm]	[mm]	[mm]	[MPa]	[MPa]	[GPa]	[%]
T2	100	180	3.2	20	19	2	0.4
T3	100	180	3	25	14	2	0.4
T4	100	180	3.4	23	22	2	0.4
T5	100	180	3.5	20	15	2	0.4
T6	100	180	2.8	25	15	2	0.4

**Table 2:** Values characteristics of sample wire T2.

Coil No/Packet No	2	Type	Circle
Size (mm)	3.2	So (mm^2^)	8.04
Lo (mm)	100	Parallel Length (mm)	100
Le	180	Rn (MPa)	20
Fm (kN)	0.146	Rp (MPa)	19
Fp (kN)	0.156	Rt (MPa)	6
Ft (kN)	0.049	End_Ex	3.56
E (GPa)	2	At (%)	3.6

**Table 3:** Values characteristics of sample wire T6.

Coil No/Packet No	6	Type	Circle
Size (mm)	2.8	So (mm^2^)	6.16
Lo (mm)	100	Parallel Length (mm)	100
Le	180	Rn (MPa)	25
Fm (kN)	0.264	Rp (MPa)	15
Fb (kN)	0.311	Rt (MPa)	6.31
Fp (kN)	0.156	E (GPa)	2
Ft (kN)	0.049	At (%)	6.3

**Table 4:** Tensile test results of the 3D-printed samples.

Sample Name	Speed (%)	Feed (%)	Weight (g)	Stress (MPa)	Strain (%)	Elongation (mm)	Standard Deviation (MPa)
V80A	60	80	8.9	9.64	0.95	1.62	0.353
V80B	60	80	9.2	9.29	1.05	1.78	0.353
V80C	60	80	9.2	9.35	1.05	1.78	0.353
V80D	60	80	9.3	9.55	0.99	1.6	0.353
V80E	60	80	9.5	10.18	1.24	2.1	0.353
V20A	20	100	9.8	7.86	0.76	1.3	0.704
V20B	20	100	9.6	8.42	0.83	1.41	0.704
V20C	20	100	9.7	8.62	0.89	1.52	0.704
V20D	20	100	9.5	8	0.94	1.6	0.704
V20E	20	100	9.8	9.64	1.05	1.78	0.704
V100A	100	100	9.7	10.36	1	1.7	0.641
V100B	100	100	9.8	9.37	0.95	1.62	0.641
V100C	100	100	9.5	9.23	0.92	1.56	0.641
V100D	100	100	9.9	8.57	0.87	1.48	0.641
V100E	100	100	10.3	9.46	0.94	1.59	0.641

**Table 5:** Individual tensile strength values for samples V20, V80, and V100.

Sample Name	Tensile Strength
-	[MPa]
V80	9.29
	9.35
	9.55
	9.64
	10.18
V20	7.86
	8
	8.42
	8.62
	9.64
V100	8.57
	9.23
	9.37
	9.46
	10.36

## Data Availability

The original contributions presented in this study are included in the article. Further inquiries can be directed to the corresponding author.
